# Mortality and Change in the Prevalence of Deep Vein Thrombosis Associated With SARS-CoV-2 P.1 Variant

**DOI:** 10.7759/cureus.26668

**Published:** 2022-07-08

**Authors:** Jose Maria Pereira de Godoy, Gleison Juliano Da Silva Russeff, Carolina Hungaro Cunha, Debora Yuri Sato, Desirée Franccini Del Frari Silva, Maria de Fatima Guerreiro Godoy

**Affiliations:** 1 Cardiology and Cardiovascular Surgery, São José do Rio Preto School of Medicine (FAMERP), São José do Rio Preto, BRA; 2 Angiology and Vascular Surgery, Clínica Godoy, São José do Rio Preto, BRA; 3 Service Ecography, Hospital de Base de São José do Rio Preto, São José do Rio Preto, BRA; 4 Occupational Therapy, São José do Rio Preto School of Medicine (FAMERP), São José do Rio Preto, BRA; 5 Occupational Therapy, Clínica Godoy, São José do Rio Preto, BRA

**Keywords:** variant, viral, deep vein thrombosis, prevalence, mortality

## Abstract

Background and objective

Thrombosis is one of the significant challenges associated with cardiovascular diseases and a prominent cause of death globally. This study aimed to determine the monthly and overall mortality rates by sex and age group in patients hospitalized with coronavirus disease 2019 (COVID-19) and the prevalence of deep vein thrombosis (DVT) in those patients. We also investigated whether the severe acute respiratory syndrome coronavirus 2 (SARS-CoV-2) P.1 variant influenced DVT.

Methods

We determined the overall prevalence of COVID-19 per sex, age, and monthly mortality using hospital data at the São José do Rio Preto School of Medicine, state of São Paulo, Brazil. Data of COVID-19 patients with DVT as determined by echo-Doppler ultrasound (EDU) were analyzed by taking two time periods into account (prior to and after the onset of the predominance of the SARS-CoV-2 P.1 variant) to evaluate whether the viral variant exerted an influence on the prevalence of DVT. Patients with COVID-19 but without DVT comprised the control group. The first period was from March 2020 to February 2021, and the second was from March to June 2021.

Results

Between March 2020 and June 2021, 6,199 patients were hospitalized with COVID-19 at our institution. Of these, 2,805 (45.25%) were women and 3,376 (54.47%) were men. Two hundred fifty-four were diagnosed with DVT based on lower limb EDU. The mean mortality rate was significantly associated with sex (38.36% for men and 27.16% for women; p=0.01). The incidence of DVT in patients with COVID-19 rose significantly from 1.6% during the first study period to 7.7% during the second study period (p=0.0001), when the P.1 variant became the predominant strain. The mortality rate was significantly higher in patients with COVID-19 and DVT (58.1%) compared to the control group (33.6%; p=0.0001).

Conclusion

Based on our findings, the incidence and prevalence of DVT increased with the predominance of P.1. viral variant. Early diagnosis and the reassessment of prophylaxis are the two most important factors to be addressed in this patient population.

## Introduction

Thrombosis represents one of the significant challenges encountered in cardiovascular diseases and a major cause of death globally. The primary goal of antithrombotic therapy is to disrupt the coagulation cascade, but there is an inherent risk of bleeding and possible failure of prophylaxis since antithrombotic therapy does not involve the inflammatory process [1]. The activation of the coagulation system by inflammation is a host-defense mechanism to avoid the dissemination of the aggressor through the bloodstream and occurs through an interaction between innate immune cells and platelets via immunothrombosis [2,3].

An autopsy study by Wichmann et al. detected deep vein thrombosis (DVT) in seven of 12 patients (58%) with coronavirus disease 2019 (COVID-19) in whom there was a suspicion of venous thromboembolism prior to death; pulmonary embolism was the direct cause of death in four patients [4]. Macrovascular and microvascular complications are associated with an increased risk of in-hospital mortality. Therefore, the early diagnosis of coagulation abnormalities in COVID-19 patients is essential for appropriate prophylaxis or antithrombotic therapy in order to improve clinical outcomes [5]. Another study detected thrombotic complications in 31% of COVID-19 patients in intensive care despite systematic thromboprophylaxis [6,7]. In another study, 30% of COVID-19 patients developed asymptomatic DVT [8]. However, a systematic routine assessment of these patients is not always performed. Hence, the prevalence and incidence of thrombotic events are probably underestimated in this patient population.

We conducted this study to determine the monthly and overall mortality rates by sex, age, and monthly prevalence of DVT at a university hospital in Brazil. The study also investigated whether the occurrence of DVT had an impact on the mortality rate of patients hospitalized with COVID-19 at the institution. We also examined whether the severe acute respiratory syndrome coronavirus 2 (SARS-CoV-2) P.1 variant influenced the prevalence of DVT.

## Materials and methods

Study design and setting

We conducted an observational study of patients who had already been treated or undergoing treatment for COVID-19 with DVT at a public hospital (Hospital de Base - São José do Rio Preto) between March 2020 and June 2021. A group of 105 consecutive patients negative for DVT served as the control group.

Inclusion and exclusion criteria

Patients were included in the study if they were diagnosed with COVID-19 at a public hospital and underwent bilateral venous echo-Doppler ultrasound (EDU) of the lower limbs to assess for DVT. Patients were excluded if they were treated in an outpatient setting.

Ethical consideration

The study received approval from the Institutional Review Board of the São Jose do Rio Preto School of Medicine, São José do Rio Preto, São Paulo, Brazil (Approval No. 4.720.521).

Analysis

The overall prevalence of COVID-19 according to sex, age, and monthly mortality based on hospital data from COVID-19 patients with DVT as determined by EDU were analyzed prior to and after the onset of the predominance of the SARS-CoV-2 P.1 variant to evaluate whether the viral variant influenced the prevalence of DVT. The first period was from March 2020 to February 2021 (when the Zeta variant was predominant), and the second was from March to June 2021 (when the Gamma variant was predominant). (Source: SIVEP-Gripe/SVS/MS/FUNFARME-São José do Rio Preto-SP-Brazil).

The mortality rate of patients with DVT was compared with the mortality in the control group during the Gamma variant period. We used descriptive statistics and comparisons by employing the Fisher’s exact test with a 5% alpha error to analyze the data.

## Results

**Figure 1:**
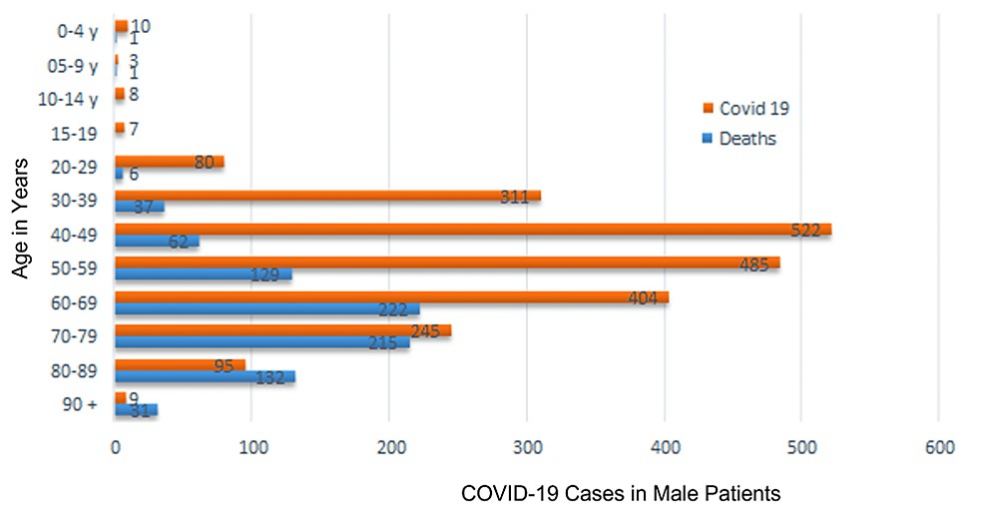
Mortality in male patients with COVID-19 by age group COVID-19: coronavirus disease 2019; y: year

**Figure 2:**
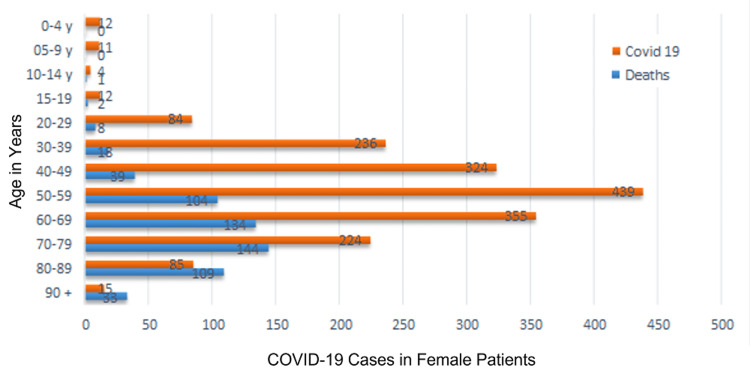
Mortality in female patients with COVID-19 by age group COVID-19: coronavirus disease 2019; y: year

**Figure 3:**
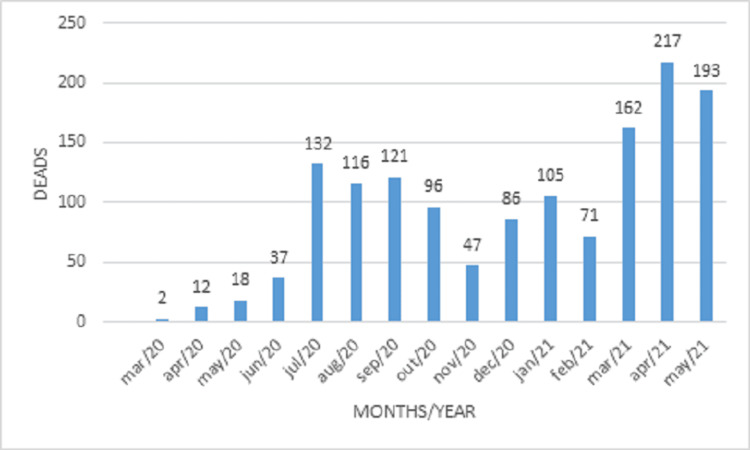
Incidence of mortality between March 2020 and May 2021 with slope

Between March 2020 and June 2021, 6,199 patients were hospitalized with COVID-19 at the university hospital affiliated with the São José do Rio Preto School of Medicine, state of São Paulo, Brazil. Of these, 2,805 (45.25%) were women and 3,376 (54.47%) were men. Two hundred fifty-four were diagnosed with DVT by lower limb EDU. A significant difference was found between the sexes regarding mean mortality rate (38.36% for men and 27.16% for women; p=0.01, Fisher’s exact test). Figure 1 and Figure 2 illustrate the mortality rate among men and women per age group, respectively. Figure 3 displays the overall monthly mortality during the period analyzed.

**Figure 4:**
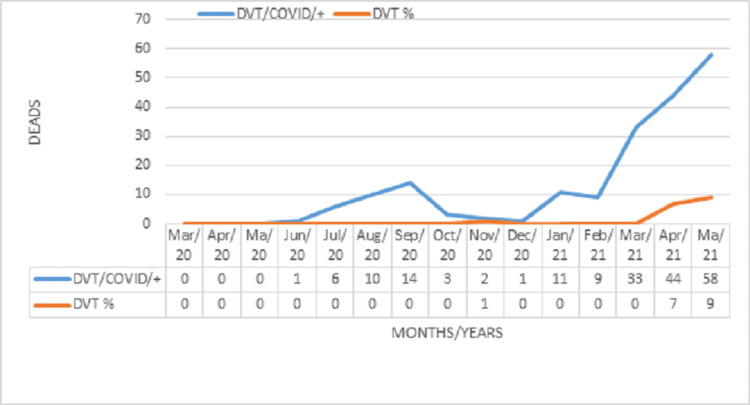
Monthly overall mortality between March 2020 and May 2021 with slope DVT: deep vein thrombosis; COVID-19: coronavirus disease 2019

Table 1 and Figure 4 demonstrate the monthly variation in the number of patients with COVID-19, those with DVT, and DVT prevalence. From June 2020 to February 2021, 3,548 patients were hospitalized for COVID-19, with 57 cases of DVT (1.6%). From March to June 2021, 2,545 patients were hospitalized for COVID-19, with 197 cases of DVT (7.7%), constituting a significant increase over the previous period (p=0.0001, Fisher’s exact test). March 2021 marked the change in viral predominance to the SARS-CoV-2 P.1. variant, accounting for more than 83% of cases in March, 97% in April, and 98% in May. The mortality rate was significantly higher among the patients with DVT (58.1%) compared to the group of 105 patients without DVT (33.6%; p=0.0001, Fisher’s exact test).

**Table 1:** Monthly number of patients with COVID-19, DVT, and the percentage of DVT among COVID-19 patients DVT: deep vein thrombosis; COVID-19: coronavirus disease 2019

Month/year	Patients with COVID-19 (n)	Patients with DVT (n)	DVT in COVID-19 (%)
March/20	6	0	0
April/20	24	0	0
May/20	76	0	0
June/20	239	1	0.41
July/20	544	6	1.1
August/20	557	10	1.7
September/20	468	14	2.99
October/20	324	3	0.92
November/20	191	2	1
December/20	372	1	0.26
January/21	498	11	2.2
February/21	355	9	2.5
March/21	633	33	5.2
April/21	624	44	7
May/21	640	58	9
June/21	648	62	7.0
Total	6,199	254	4.0

## Discussion

The present study reports the evolution of DVT associated with COVID-19 and identifies an increase in the prevalence of DVT with the predominance of the P.1 variant and an increase in mortality in these patients. Two peaks were found: July to September 2020 and March to June 2021. Moreover, the mortality rate was higher among men than women.

More than 1,300 patients with COVID-19 were evaluated with a clinical suspicion of DVT, 254 of whom had positive EDU findings. Approximately 150-200 patients with COVID-19 are admitted daily to intensive care units at our institution, suggesting that the prevalence is underestimated. The evaluations were based on D-dimer levels and clinical assessments of these patients. The positivity for DVT was initially 25-30%, which increased to 45-50%, suggesting that many patients were undiagnosed.

Identifying biochemical markers of thrombosis and an evaluation of D-dimer levels point to worrisome data, as the difference in mortality in patients with and without DVT is significant. One study has reported that the mortality rate was >50% higher among patients with D-dimer levels of 3,000 ng/ml and DVT and less than 30% among those without thrombosis [9]. Therefore, the indication for EDU should not be based merely on D-dimer findings in these patients. The mortality rate was higher among those with DVT than those without DVT. Therefore, DVT is a factor associated with mortality in patients with COVID-19 [10].

Prophylaxis has failed at unacceptable rates in intensive care units. COVID-19 patients received routine prophylaxis with enoxaparin and, more recently, rivaroxaban. This failure rate suggests a change in the physiopathology, indicating a need for a better prophylactic option. Immunothrombosis, a broader aspect in the physiopathology of thrombosis in COVID-19, involves the inflammatory process, immunity, and the coagulation cascade. Therefore, novel alternatives are needed for prophylaxis with respect to DVT, which is linked to a two-fold increase in mortality rate in COVID-19 patients. Better prophylaxis for DVT and early diagnosis and treatment of thrombosis constitute the two current major challenges for vascular surgeons.

Our study had a serious limitation. We did not assess other relevant COVID-19 comorbidities including associated cardiomyopathies, obesity, and diabetes. Including these comorbidities would have made our findings more robust.

## Conclusions

We conducted this study to determine mortality rates by sex and age group in patients hospitalized with COVID-19 and the prevalence of DVT, a frequent complication in COVID-19 patients. Based on our findings, the incidence and prevalence of DVT increased with the predominance of P.1 viral variant. Early diagnosis and the reassessment of prophylaxis are critical factors in treating patients with COVID-19 and DVT.

## References

[1] Stark K, Massberg S (2021). Interplay between inflammation and thrombosis in cardiovascular pathology. Nat Rev Cardiol.

[2] Engelmann B, Massberg S (2013). Thrombosis as an intravascular effector of innate immunity. Nat Rev Immunol.

[3] Nicolai L, Leunig A, Brambs S (2020). Immunothrombotic dysregulation in COVID-19 pneumonia is associated with respiratory failure and coagulopathy. Circulation.

[4] Wichmann D, Sperhake JP, Lütgehetmann M (2020). Autopsy findings and venous thromboembolism in patients with COVID-19: a prospective cohort study. Ann Intern Med.

[5] Gąsecka A, Borovac JA, Guerreiro RA, Giustozzi M, Parker W, Caldeira D, Chiva-Blanch G (2021). Thrombotic complications in patients with COVID-19: pathophysiological mechanisms, diagnosis, and treatment. Cardiovasc Drugs Ther.

[6] Klok FA, Kruip MJ, van der Meer NJ (2020). Incidence of thrombotic complications in critically ill ICU patients with COVID-19. Thromb Res.

[7] Klok FA, Kruip MJ, van der Meer NJ (2020). Confirmation of the high cumulative incidence of thrombotic complications in critically ill ICU patients with COVID-19: an updated analysis. Thromb Res.

[8] Cui S, Chen S, Li X, Liu S, Wang F (2020). Prevalence of venous thromboembolism in patients with severe novel coronavirus pneumonia. J Thromb Haemost.

[9] Godoy JMP, da Silva MOM, Santos HA, Silva AFV, Russeff GJS (2022). Mortality, deep vein thrombosis and D-dimer levels in patients with COVID-19 (IN PRESS). Cor et Vasa.

[10] Pereira de Godoy JM, Russeff GJ, Cunha CH (2022). Increased prevalence of deep vein thrombosis and mortality in patients with Covid-19 at a referral center in Brazil. Phlebology.

